# Transition From Open and Laparoscopic to Robotic Partial Nephrectomy: Learning Curve and Outcomes

**DOI:** 10.7759/cureus.51646

**Published:** 2024-01-04

**Authors:** Shritosh Kumar, Brusabhanu Nayak

**Affiliations:** 1 Urology, All India Institute of Medical Sciences, New Delhi, IND

**Keywords:** open, laparoscopy, renal cell carcinoma, robot, partial nephrectomy

## Abstract

Introduction

Partial nephrectomy (PN) is the current standard of care for patients with T1 renal tumors, and there has been a shift from an open and laparoscopic to a robot-assisted approach. The definition of the learning curve for robot-assisted PN (RAPN) is unclear, and various studies have identified warm ischemia time (WIT), perioperative complications, and surgical margins as the defining parameters for the assessment of improvement in these outcomes over time. The objective of this study was to evaluate the learning curve of a newly trained urologist for RAPN when comparing both open and laparoscopic approaches.

Methods

This study included 52 patients who underwent PN by open, laparoscopic, and robotic methods performed by a single, newly trained urologist over a period of seven years. Basic demographic and perioperative data were collected, and the learning curve was compared between the three approaches.

Results

Baseline parameters were similar for open (n = 15), laparoscopic (n = 12), and robotic (n = 25) PN except for tumor size and nephrometry score, which were higher in the open group (p = 0.000). Operative time was significantly longer in the robotic approach (180 minutes; p = 0.05), and blood loss was greater in the open group (450 mL; p = 0.000). Median WIT was 25 minutes; significant complications (Clavien Dindo ≥II) and positive surgical margins were 12% and 0%, respectively, in the robotic arm. Preoperative imaging and final histopathology data showed larger tumors being operated on, preferably by an open method, than laparoscopic and robotic PN (6.3 cm vs. 3.4 cm; p = 0.000). More open and laparoscopic procedures (n = 12, 10) were performed during the initial 26 cases, with a later transition to robot-assisted PN (n = 21) in the next 26 cases. None of the parameters showed improvement in the latter half, while operative time showed an increase (150 vs. 180 minutes; p = 0.045).

Conclusion

The learning curve becomes similar across three defined parameters, i.e., WIT, perioperative complications, and positive surgical margins, after performing a minimum of 25 RAPNs when compared to open and laparoscopic approaches. However, operative duration continues to improve and may take longer to become comparable. A newly trained urologist can safely perform RAPNs even with a small number of cases, especially those who have been previously trained for open and laparoscopic cases.

## Introduction

Kidney cancer accounted for 2.2% of all cancers, with 430,000 cases and 179,000 deaths worldwide in 2020 [1]. The incidence of localized renal cancers has increased in the last decade, mainly owing to more frequent imaging [2]. Partial nephrectomy (PN) was recognized in 2006 as an alternative to radical nephrectomy and is currently recommended for localized T1 renal tumors and the preferred approach for T2 tumors in patients with a solitary kidney or chronic kidney disease (CKD) [3]. Studies have compared open, laparoscopic, and robot-assisted PN (RAPN) and confirmed that laparoscopic and robotic approaches are associated with equivalent oncological outcomes, a shorter length of hospital stay, and lower blood loss [4,5]. The current evidence comparing the three approaches includes data from multiple surgeons and centers [6]. However, the learning experience for new urologists starting with open and laparoscopic methods and later performing robotic PN is infrequently reported. We compared open PN (OPN), laparoscopic PN (LPN), and RAPN performed by a newly trained surgeon over seven years and reported the perioperative outcomes. The learning curve was analyzed for RAPNs with respect to warm ischemia time (WIT), positive surgical margins, and surgical complications.

## Materials and methods

Objectives

The primary objective of the study was to compare the learning curve (defined as the number of cases taken over time where improvement is observed in terms of WIT, positive surgical margins, and complications) for RAPN vs. OPN and LPN. Secondary objectives were to compare the initial half (Group 1: first 26 cases) with the latter half (Group 2: next 26 cases) for the number of cases done using the three approaches and perioperative outcomes.

Data collection

Patients who underwent PN by all three approaches, i.e., OPN, LPN, and RAPN, in our tertiary care center between June 2015 and February 2022 by a newly trained single surgeon were included in this study. Retrospectively, data was collected from the central database of the institute in the urology department, and 52 consecutive cases were included. A sample size calculation was not needed owing to the retrospective nature of the study. Patients who were operated on by other surgeons with prior experience were excluded. Procedure selection was based on various factors, including tumor complexity, renal function, and the surgeon's preference. Preoperative data included age, gender, tumor location, and size based on imaging, RENAL nephrometry score, comorbidities, and baseline blood parameters, including hemoglobin and serum creatinine. Operative parameters included WIT, blood loss, total operative time, pelvicalyceal repair, and/or double J stent use. Postoperative data included length of hospital stay, discharge of hemoglobin and creatinine, and complications within 30 days of surgery. Complications, if any, were reported according to the Clavien-Dindo grading. A dedicated uropathologist reviewed histopathology for tumor type and margin status.

Technique

The OPN was performed with the patient in a lateral decubitus position and the flank positioned overlying the table break for flexion. A flank approach (transcostal) was used to gain access, with or without rib resection. Muscle layers were divided to access the kidney retroperitoneally, and the renal hilum was identified after adequate dissection. Both the renal artery and vein were identified and isolated with vascular loops, and the artery was clamped with a vascular clamp. Intraoperative ultrasound was used whenever identification of mass or margins was difficult, on a case-to-case basis. The renal mass was resected with a sharp dissection, and if any pelvicalyceal system breach was found, it was closed using a nonabsorbable monofilament suture. The placement of the double-J stent was at the discretion of the surgeon. A drain was placed in all three approaches. The LPN was done with the patient in a modified flank position and with four ports placed transperitoneally. Dissection was similar to an open approach with a laparoscopic vascular clamp used for clamping the renal artery. After removing the mass, parenchyma was approximated using v-lock sutures with hemostat cellulose bolsters clipped with Weck® Hem-o-lok® clips (Teleflex Incorp., Wayne, PA, USA). The RAPN was performed using the Da Vinci Xi robotic system (Intuitive Surgical Inc., Sunnyvale, CA, USA). Transperitoneal access was used for port placements and dissection, and clamping and suturing were done in a similar pattern as LPN.

Statistical analysis

Data were processed using SPSS Statistics version 25.0 (IBM Corp., Armonk, NY, USA). Continuous data were expressed as mean±SD for normally distributed data or median (interquartile range) for skewed data. A p-value of <0.05 was considered statistically significant. Categorical variables were compared using a Chi-square test, and continuous variables were compared using a one-way ANOVA.

## Results

Of the 52 patients included in the study, 15 OPN, 12 LPN, and 25 RAPN were performed by a single surgeon over seven years. Out of 52, the first 26 surgeries were done by September 2019 and included 12 OPNs, 10 LPNs, and 4 RAPNs. Most RAPNs were performed in the latter 26 cases (n = 21).

There were no significant differences between the three approaches in terms of age, gender, tumor laterality, preoperative hemoglobin, or creatinine levels. Larger and more complex tumors were more frequently operated on by an OPN approach (OPN: 6.6±2.3cm, LPN: 3.8±0.99 cm, RAPN: 3.9±1.17cm, p = 0.000). The mean tumor size was 4.6 cm. The nephrometry score was calculated with the RENAL scoring system [7]. Patients undergoing OPN had higher nephrometry scores (p = 0.000). More patients had associated diabetes (15%) or hypertension (35%), but they were distributed similarly across all three approaches (Table 1).

**Table 1:** Preoperative demographic data Values in bold suggest a statistically significant difference where p≤0.05. OPN: Open partial nephrectomy, LPN: Laparoscopic partial nephrectomy, RAPN: Robot-assisted partial nephrectomy, PN: Partial nephrectomy, UP: Upper pole, IP: Inferior pole, LP: Lower pole, Ca: Cancer, Hb: Hemoglobin

Variable	OPN (n=15)	LPN (n = 12)	RAPN (n = 25)	p-value	Total PN (n = 52)
Age (mean±SD)	45±11.71	49.2±14.1	48.1±10.7	0.847	47.8±11.56
M/F	9/6	9/3	17/8	0.707	31/15
Tumor laterality (right/left)	7/8	4/8	10/15	0.780	21/31
Tumor size (cm) (imaging) (mean±SD)	6.6±2.3	3.8±0.99	3.9±1.17	0.000	4.6±1.9
Pole (UP/IP/LP)	4/5/6	4/3/5	10/10/5	0.583	18/18/16
RENAL nephrometry score (mean±SD)	7.0±1.0	5.2±0.97	4.76±1.23	0.000	5.4±1.47
Co-morbidities				0.452	
1. Diabetes mellitus	2	1	4		7
2. Hypertension	4	4	8		16
3. Chronic kidney disease	0	1	0		1
4. Abdominal tuberculosis	0	0	2		2
5. Chronic liver disease	0	0	1		1
6. Ca Colon	1	0	0		1
7. Chronic obstructive pulmonary disease	0	0	2		2
8. Hepatitis C	0	1	0		1
9. Hypothyroidism	0	0	2		2
10. Irritable bowel syndrome	0	0	1		1
Baseline Hb (mean±SD)	12.4±2.26	13.12±1.78	13.02±1.72	0.698	12.85±1.84
Baseline creatinine (mean±SD)	0.78±0.33	1.26±1.22	0.80±0.22	0.115	0.89±0.59

Intraoperatively, WIT was shorter in the OPN (18 minutes vs. 25 minutes in the LPN and RAPN), but this difference was not significant (p = 0.054). In eight cases, the collecting system was opened and subsequently repaired. A double-J stent was used in only one case of OPN. One case of LPN was converted to open due to uncontrollable bleeding. Blood loss was higher in OPN (OPN: 450 mL, LAPN: 150 mL, RAPN: 150 mL, p = 0.000). Operative time was the longest in the RAPN group (OPN: 120 minutes, LPN: 150 minutes, RAPN: 180 minutes) (Table 2).

**Table 2:** Perioperative data Values in bold suggest a statistically significant difference where p≤0.05. OPN: Open partial nephrectomy, LPN: Laparoscopic partial nephrectomy, RAPN: Robot-assisted partial nephrectomy, PN: Partial nephrectomy

Variable	OPN (n = 15)	LPN (n = 12)	RAPN (n = 25)	p-value	Total PN (n = 52)
Clamping (Y/N)	14/1	12/0	25/0	0.419	51/1
Warm ischemia time minutes (median, IQR)	18 (13-25)	25 (19-26)	25 (20-30)	0.054	25 (18-29)
PCS status (opened/not opened)	4/11	2/10	2/23	0.282	8/44
Stent use (Y/N)	2/13	0/12	0/25	0.128	2/50
Conversion to open (Y/N)	0/15	1/11	0/25	0.324	1/51
Blood loss in mL (median, IQR)	450 (350-500)	150 (50-350)	150 (105-200)	0.000	200 (115-350)
Operative time in minutes (median, IQR)	120 (120-180)	150 (135-165)	180 (150-195)	0.050	150 (150-180)

The median length of stay, hemoglobin, and serum creatinine at the time of discharge were similar for all three approaches. One case in OPN leaked and was managed with a double J stent, percutaneous nephrostomy, and cyanoacrylate glue, which was reported earlier [8]. Complication rates were also similar between approaches (OPN: urine leak, LPN: blood transfusion, RAPN: one pneumothorax, two respiratory complications). Pathological evaluation of the tumor specimen revealed that most tumors were clear cell renal cell carcinoma (RCC) (63%), and papillary RCC (15%). Others included angiomyolipoma (AML, 8%), chromophobe RCC (4%), cystic neoplasm of low malignant potential (4%), oncocytoma (4%), and leiomyoma (2%). The histological size also differed significantly with larger tumors being operated on using an open approach (OPN: 6.3 cm, LPN: 3.4 cm, RAPN: 3.4 cm, p = 0.000). Positive surgical margins were histologically seen in two patients who underwent OPN (both AML) and one case in LPN (papillary RCC) (Table 3).

**Table 3:** Postoperative and histopathology data Values in bold suggest a statistically significant difference where p≤0.05. OPN: Open partial nephrectomy, LPN: Laparoscopic partial nephrectomy, RAPN: Robot-assisted partial nephrectomy, PN: Partial nephrectomy, AML: Angiomyolipoma, RCC: Renal cell carcinoma

Variable	OPN (n = 15)	LPN (n = 12)	RAPN (n = 25)	p-value	Total PN (n = 52)
Postoperative hospital stays median (IQR)	6 (5-6)	5 (4-5)	5 (4-6)	0.056	5 (4-6)
Discharge Hb (mean±SD)	10.48±1.0	11.73±1.80	11.34±1.65	0.131	11.21±1.58
Discharge creatinine (mean±SD)	0.90±0.54	1.37±1.29	1.11±0.29	0.315	1.11±0.67
Urine leakage	1	0	0	0.235	1
Complications (Clavien-Dindo): None/grade I/grade II/grade III/grade IV (within 30 days)	14 0 1 0	11 1 0 0	22 0 1 2	0.845	47 1 2 2
Histological size (cm) (mean±SD)	6.31±2.25	3.44±0.79	3.39±0.92	0.000	4.34±2.11
Histopathology				0.454	
Clear cell RCC	9	9	15		33
Papillary RCC	2	2	4		8
AML	2	0	2		4
Cystic neoplasm of low malignant potential	1	0	1		2
Oncocytoma	1	0	1		2
Chromophobe RCC	0	0	2		2
Leiomyoma	0	1	0		1
Positive margins (Y/N)	(2/13) 2 AML	(1/11) 1 papillary RCC	(0/25)	0.132	(3/49)

When comparing the first 26 cases (Group 1) with the latter 26 (Group 2), more robotic cases (8% vs. 40%) were performed during the latter part of the surgeon's career. Interestingly, the WIT remained similar between the two groups. The median blood loss and operative stay also remained similar between the two groups. However, operative time was significantly longer in the latter half, owing to a greater number of robotic cases being performed (Table 4).

**Table 4:** Comparison between the first half (Group 1) vs. the latter half cases (Group 2) Values in bold suggest a statistically significant difference where p≤0.05. OPN: Open partial nephrectomy, LPN: Laparoscopic partial nephrectomy, RAPN: Robot-assisted partial nephrectomy, WIT: Warm ischemia time, CD: Clavien Dindo

Variable	Group 1 (n = 26)	Group 2 (n = 26)	p-value
OPN/LPN/RAPN	12/10/4	3/2/21	0.000
Tumor size (cm) (Imaging) (mean±SD)	4.87±2.28	4.29±1.43	0.247
RENAL nephrometry score (mean±SD)	5.82±1.44	5.09±1.47	0.109
WIT in minutes (median, IQR)	25 (15-26.25)	25 (20-30)	0.155
Blood loss mL (median, IQR)	275 (137.5-462.5)	150 (110-250)	0.072
Time in minutes (median, IQR)	150 (120-180)	180 (150-210)	0.045
Postoperative hospital stays median (IQR)	5 (4-6)	5 (4-6)	0.822
Complications: None/CD≥II	24/2	23/3	0.638
Positive margins (Y/N)	3/23	0/26	0.235

## Discussion

For small, localized T1 renal masses, PN is the current treatment of choice and is equivalent in terms of oncological outcomes and progression-free survival compared to radical nephrectomy (RN) [9]. For T2 tumors, European guidelines recommend PN if technically feasible, especially in the setting of a solitary kidney, CKD, or bilateral renal tumors [3]. Studies comparing OPN vs. LPN have found no difference in both progression-free survival and overall survival [10]. Another study comparing OPN and RAPN found RAPN to be associated with fewer complications, blood transfusion, and shorter hospital stays [4]. Few published studies to date have compared all three approaches [11]. The present study compares all three surgical approaches for PN performed by a single urologist at a tertiary care center. The urologist was a newly trained surgeon and started with open and laparoscopic cases first. The data has been collected consecutively for 52 patients operated on over seven years, highlighting subsequent experience with the procedure and differences in case selection for various methods. The surgeon's comfort level with both LPN and RAPN grows with the number of cases performed, and this study reflects a similar transition from OPN and LPN to RAPN.

In our series, the mean tumor size was 4.6±1.9 cm, substantiating the role of PN for T1 lesions. However, OPN was performed for a mean tumor size of 6.6±2.3 cm. Although most OPNs (12 out of 15) were performed in the first half of 52 cases, three cases were performed later when the surgeon was comfortable with RAPN. These three cases had a large, complex tumor, and a preoperative decision was made to choose an open approach, reflecting the importance of OPN in select patients even in the era of RAPN. Intraoperative blood loss was significantly higher in the OPN group, similar to previously reported findings by Masson-Lecomte et al. [12]. Additionally, total intraoperative time was higher for RAPN in our study, which has also been a common criticism of robotic procedures in the past. But this could well be due to a significant learning curve with the robot, and future comparisons are needed to see any further decrease. The WIT was shorter for OPN (18 minutes) compared to LPN (25 minutes) and RAPN (25 minutes), but this difference was statistically insignificant. The WIT has already been shown to be shorter in OPN than in LPN [13,14].

Experiences of single surgeons transitioning from LPN to RAPN have been published with similar perioperative outcomes [15]. Another study by Klaassen et al. compared all three modalities and found that RAPN was associated with fewer complications than OPN and LAPN [11]. Complication rates were similar in our study; however, RAPN did have less total blood loss than OPN. Postoperative hospital stays have been previously shown to be shorter in RAPN, but our study could not reflect the same results. Our hospital is a tertiary care center in a low- and middle-income country, and patients travel far for treatment. Discharge of these patients is dependent on multiple factors, and thus hospital stay was higher even in the robotic and laparoscopic arm. The median stay was five days for both LPN and RAPN and six days for OPN.

Positive surgical margins have been documented in 2% to 8% of PN specimens [16]. We had a 7% positive margin rate, although two were preoperatively suspected to have AML based on imaging, and margin control was not the primary concern in these cases. Only one case (2%) had a positive margin, which eventually was determined to be a papillary RCC, and the patient was started on tyrosine kinase inhibitors.

The learning curve concerning WIT, positive surgical margins, and complications did not show any significant improvement after performing 25 RAPNs in our study. The WIT was 25 minutes. A previous study published by our center with the inclusion of multiple surgeons and more than 20 years of experience showed ischemia times of 18.9 vs. 24.1 vs. 20.6 minutes between OPN, LPN, and RAPN, respectively [17]. Another study explored the effect of increasing surgical experience on WIT and complications. They concluded that improvement in ischemia times occurs for up to 150 cases, after which it reaches a plateau [18].

Our study compared all three approaches simultaneously performed by a newly trained urologist. In seven years with 52 cases, the mean ischemia time was still similar. More importantly, the surgical margins and complication rates were also similar. Since this is a retrospective analysis, it does bring inherent limitations to the study. The sample size was small, and tumor size and complexity were higher in the OPN group. The non-inclusion of the experience of the assistant and the department, which may affect the perioperative parameters, is another limitation. A single-surgeon analysis may minimize a few confounders. The comparison of all three groups reflects the actual real-world clinical data at a tertiary center, along with patient and procedure selection based on the surgeon's experience, which are some of the potential strengths of this study.

## Conclusions

Partial nephrectomy is the current treatment of choice for T1 renal masses, and open, laparoscopic, or robotic approaches are all reasonable options for treatment. The transition from OPN and LPN to RAPN for a newly trained urologist is not associated with any increase in positive surgical margins or complications, except for longer operative times. The RAPN is a safe approach for small renal masses, and the learning curve remains similar with respect to ischemia time across the three approaches.
